# Multidimensional pruritus assessment in hemodialysis patients

**DOI:** 10.1186/s12882-019-1234-0

**Published:** 2019-02-06

**Authors:** Nese Altınok Ersoy, İmatullah Akyar

**Affiliations:** Medical Nursing Department, Hacettepe University Faculty of Nursing, Sihhiye/Ankara, Turkey

**Keywords:** Hemodialysis, Nursing, Pruritus, Chronic renal failure, 5-D itch scale

## Abstract

**Background:**

Pruritus is a distressing, life-limiting symptom in chronic renal failure, affecting 40% of patients. This study aimed to determine uremic pruritus prevalence and investigate the multidimensional impact on hemodialysis patients.

**Methods:**

This descriptive study was performed between March and June 2016. The study included 181 patients undergoing hemodialysis session, who reported pruritus in the prior month. Data were collected using the 5-D Itch Scale, which assesses pruritus based on 5 dimensions, i.e., degree, duration, direction, disability, and distribution, with a total score ranging from 5 (no itching) to 25 (maximum severity).

**Results:**

Pruritus prevalence was 49.3%. Patients had a mean score of 13.97 ± 4.11 (moderate severity). The daily duration was 6–12 h (40.3%), with direction “a little bit better but still present” (38.7%) and distribution on the “back, upper arms, chest, and abdomen.” Patients sleep, social life/leisure time, housework and errand were impacted “occasionally”. The score was higher in patients aged ≥65 years, those on hemodialysis for ≥15 or more years, and those undergoing afternoon hemodialysis. The duration of itching was significantly shorter in employed patients.

**Conclusion:**

Assessment and management of itching symptoms in chronic renal failure are a clinical priority both for patients and for health care professionals. The results of this study highlight the importance of multidimensional assessment and support the need for development of standardized and patient-specific symptom management.

## Background

Itching, an unpleasant sensation of the skin, causing a desire to scratch, is a bothersome and unpleasant symptom in chronic renal failure (CRF) patients and has the highest prevalence among cutaneous manifestations in renal disease [1, 2]. The prevalence of uremic pruritus varies between 10 and 77% [3].

Uremic pruritus has a multifactorial etiopathogenesis, and is associated with physical disability, impaired quality of life, and limitations in activities of daily living [4, 5]. Uremic pruritus is strongly associated with physical and mental limitations, insomnia, and chronic fatigue; discomfort, embarrassment/isolation, and secondary skin changes due to scratching of lesions; and anger, anxiety, and depression [6–8].

Despite the high prevalence and life changing effects, multidimensional symptom evaluation and the main effects on patient life remain poorly characterized, as most research has focused on severity and direction. Multidimensional itch assessment should be conducted to assess and manage itching symptom in clinical environment due to multifactorial nature. Multidimensional approach for pruritus includes measuring degree, duration, direction, impact on activities of daily life, and location of pruritus. As further multifaceted paradigm of itching taken into consideration, assessment of care and treatment success also be evaluated within numerous dimensions. This time and individual sensitive approach also helps to screen and record the changes over time [9].

An important role of health care professionals is recognition and management of pruritus symptoms in hemodialysis patients, as well as determination of the effect on quality of life, and assessment, evaluation, and treatment of symptoms using a biopsychosocial approach [10]. Hence, this study aimed to determine the prevalence of uremic pruritus and to describe the multidimensional symptom experience in hemodialysis patients, according to duration, degree, direction, disability, and distribution, as well as to determine the associations between pruritus, patient clinical features, and dialysis parameters.

## Methods

### Study design

This study was conducted with a descriptive design to determine pruritus prevalence in hemodialysis patients and the associations between its presence and the effect on clinical features and dialysis parameters.

The study will address the following research questions:

- What is the prevalence of uremic pruritus?
- What are the characteristics of pruritus symptoms in CRF patients according to degree, duration, direction, disability/impact on activities of daily living, and distribution?
- What are the associations between pruritus and patient clinical features and dialysis parameters?

### Sample and setting

This study was performed in three university hospital dialysis centers in Ankara, Turkey. Hemodialysis patients were enrolled using the following inclusion criteria: older than 18 years of age, presence of pruritus in the prior month, and ability to communicate. Patients receiving pharmacological treatment for pruritus and those with liver diseases (e.g., hepatitis) were not considered for the study. Sample size calculation was performed using NCSS and PASS software, with 90% power, 0.05 alpha, and 0.50 effect size. Sample size was determined as 172 patients.

Of 391 patients enrolled between March and June 2016, the study considered 181, using a random sample selection method. Eleven patients declined to participate, 10 received nighttime dialysis, 2 had cognitive disorders, 1 was younger than 18 years, and 186 did not report itching.

### Instruments

Sociodemographic data were collected and the 5-D Itch Scale score was determined. A data worksheet was created by reviewing the literature [3, 7, 11, 12] and included questions on age, sex, height and weight, educational level, occupation, illness duration, stage of CRF, comorbidities, medications, and dialysis parameters. The dialysis parameters included treatment duration; session duration; speed (blood flow rate); adequacy (Kt/V, where k: dialyzer clearance of urea in a single treatment, in mL/min; t: time; V: volume of body fluid); itching before, after, and during a hemodialysis session; and the day of itching after hemodialysis treatment.

The 5-D Itch Scale was developed by Elman et al. in 2010. The scale assesses duration, degree, direction, distribution, and disability associated with itching in the prior 2 weeks. The total score ranges between a minimum of 5 points (no itching) and maximum of 25 points (maximum severity). The duration, degree, direction, and disability were scored from 1 to 5 points. The score for the disability dimension, with 4 subsections (sleep, social/leisure, housework/errands, work/school) was obtained from the mean of the total score for the 4 subsections. The score for distribution was obtained by examining 16 body regions according to the number of affected body parts, with a maximum score of 5 points, 0–2: 1 point, 3–5: 2 points, 6–10: 3 points, 11–13: 4 points, and 14–16: 5 points [7]. Altınok Ersoy and Akyar conducted a validation and reliability study of the scale in Turkish, with Cronbach’s Alfa coefficient 0.608 (*p* > 0.05) [13].

### Data collection procedure

A pilot study was conducted in March 2016, using the data worksheet and 5-D Itch Scale in 20 patients. A researcher performed a face-to-face interview during a hemodialysis session at 1 of the dialysis centers. The data from the pilot study were used for sample size calculation (power analysis). Pilot testing also helped to assess the intelligibility and practicality of the research design and data collection forms. As no changes were made in the pilot study protocol, the data of these 20 patients were included in the study. In addition to these 20 patients, the data sheet and 5-D Itch Scale were administered to 161 patients by a researcher during a hemodialysis session in 3 dialysis centers between March and June 2016. Informed consent was obtained from the patients to participate in the research. Data collection forms were administered over 11–12 min in total. Data collection was completed for 181 patients.

### Data analysis

Analysis was performed with SPSS software, Version 23 (IBM, Armonk, NY, USA). For Frequency (number, percentage) was used to report categorical variables, with mean and standard deviation for continuous variables.

Analysis of data obtained from 181 patients (*n* > 30) was carried out with parametric tests [the skewness coefficient of 0.718 (0.130/0.181) was between − 1.96 and + 1.96]. An independent-samples t-test was used for testing the difference between 2 independent groups; for more than 2 independent groups, unidirectional analysis of variance was used. Linear relationships between 2 continuous variables were tested with Pearson’s correlation coefficient, followed by Tukey’s multiple comparisons test [12]. A *p*-value < 0.05 was considered significant.

### Ethical considerations

Ethical (IRB) approval was granted by the ethics committee of the Hacettepe University (Hacettepe University Ethical Committee, approval # GO 15/470–20). From the administration of the Baskent University Hospital written permission was granted to conduct the study in three dialysis centers (Umitkoy, Yenikent and Cigdem Dialysis Centers within the structure of Baskent University Hospital). Signed informed consent was obtained from patients.

## Results

### Sample characteristics

Mean patient age was 56.9 ± 15.3 years, with 34.8% aged 50–65; 52.5% were male, 76.2% were primary school graduates, and 95.5% were unemployed. Nearly half of the patients (38.1%) had a diagnosis of CRF for ≥15 years (13.5 ± 6.5 years). The mean dialysis vintage was 12.2 ± 6.5 years, ranging from 1 to 25 years, with 59.1% of patients receiving hemodialysis for ≥11 years. Hypertension was present in 53 and 27% had diabetes mellitus. All patients received hemodialysis 3 times per week; 53.6% received treatment in a morning session, with 46.4% in an afternoon session. The mean duration of a hemodialysis session was 4.08 ± 1.02 h, with mean Kt/V for hemodialysis adequacy of 1.3 ± 0.16, and mean hemodialysis speed of 620 ± 150 ml/min.

### Itching characteristics

Of 181 patients evaluated, itching was observed in 49.3%; 86.7% reported itching before the hemodialysis session, 72.9% during hemodialysis, and 49.7% after hemodialysis.

The daily duration of itching was 6–12 h in 40.3% of patients, whereas 1.1% had day-long itching. The itch degree was described as moderate (40.3%), mild (30.4%), severe (28.2%), or unbearable (2%). The direction of itching in the prior month was reported as “a little bit better, but still present” in 38.7% of patients and “getting worse” in 5%.

The impact of itching on sleep was described as “delay in falling asleep and occasionally wakes me up at night” by 50.8% of the patients. Disability for social life/leisure time was described as “occasional” in 38.1%, with disability for housework and errands also described as “occasional” in 38.7%; disability for work/school was described as “not applicable” in 95.6%. The distribution of itching involved 6–10 anatomical regions in 65.2%. The most commonly reported anatomical regions were the back, upper arm, chest, and abdomen; the least reported anatomical regions were the palms, soles, and face/head (Table 1).

**Table 1:** Descriptive Findings of 5-D Itch Scale (*n* = 181)

5-D Itch Scale Dimension	*N*	%	Mean ± SD	Median (Min-Max)
Duration				
Less than 6 h/day	43	23.8	2.2 ± 0.9	2 (1–5)
6–12 h/day	73	40.3		
12–18 h/day	45	24.9		
18–23 h/day	18	9.9		
All Day	2	1.1		
Degree*				
Mild	55	30.4	3.0 ± 0.8	3 (2–5)
Moderate	73	40.3		
Severe	51	28.2		
Unbearable	2	1.1		
Direction				
Completely resolved	0	0	3.3 ± 0.8	3 (2–5)
Much better but still present	36	19.8		
Little bit better but still present	70	38.7		
Unchanged	66	36.5		
Getting worse	9	5.0		
Distribution				
0–2 region	1	0.6	2.9 ± 0.6	3 (1–5)
3–5 region	42	23.2		
6–10 region	118	65.2		
11–13 region	16	8.8		
14–16 region	4	2.2		
Disability **–** *Sleep*				
Never affects sleep	6	3.3	3.8 ± 0.9	4 (1–5)
Occasionally delays falling asleep	17	9.4		
Frequently delays falling asleep	29	16.0		
Delays falling asleep and occasionally wakes me up at night	92	50.8		
Delays falling asleep and frequently wakes me up at night	37	20.5		
Disability **-** Social/ Leisure*				
Not applicable	4	2.2	2.5 ± 0.8	4 (1–4)
Never	23	12.7		
Rarely	62	34.3		
Occasionally	69	38.1		
Frequently	23	12.7		
Disability **-** Housework/Errands*				
Not applicable	6	3.3	2.4 ± 0.8	3 (1–4)
Never	26	14.4		
Rarely	69	38.1		
Occasionally	70	38.7		
Frequently	10	5.5		
Disability - Work/School*				
Not applicable	173	95.6	1.6 ± 0.7	0 (1–3)
Never	4	2.2		
Rarely	3	1.2		
Occasionally	1	0.6		
Frequently	8	0.4		

*Always “not present” excluded from the sample

*Degree “not present” excluded from the sample

The longest itching duration (mean 2.4 ± 0.9 h), highest degree (mean 3.2 ± 0.8), worst direction, and the highest total 5-D Itch Scale score (3.6 ± 0.7) were observed in patients aged > 65 years. The total score was higher in patients whose duration of CRF and hemodialysis treatment was > 15 years (*p* < 0.05). Disability in primary school graduates and itching time score averages in unemployed patients were higher than those in other groups (*p* < 0.05). The mean afternoon session score was higher than the mean morning score, and statistically significant differences were observed between degree, direction dimension, and scale total scores (*p* < 0.05) (Table 2).

**Table 2:** Distribution of 5-D Itch Scale Total or Dimension Scores Attributed to Patients Clinical Features

	*N*	Mean ± SD	Statistical analysis
Gender and Scale Total Score			
Male	95	14.9 ± 2.9	p: 0.19 t: −1.305
Female	86	15.5 ± 2.8	
Age and Scale Total Score^a^			
20–49	60	14.9 ± 3.1	p: 0.04 F: 3.058
50–65 (2)^a^	63	14.7 ± 2.5	
66–88 (3)^a^	57	15.9 ± 2.9	
Graduation Level and Disability Dimension Score^b^			
Primary school (1)^b^	138	3.9 ± 0.9	p: 0.00 F: 6.438
Secondary school	17	3.5 ± 1.2	
High school	15	3.7 ± 0.6	
University and Post-Graduate (4)^b^	11	2.6 ± 1.2	
Employment Status and Duration Dimension Score			
Unemployed	173	2.3 ± 0.9	p: 0.01 t: 1.485
Employed	8	1.7 ± 0.4	
CRF Diagnosis Duration and Scale Total Score^c^			
1–10 Year	62	15.0 ± 3.0	p: 0.01 F: 4.08
11–15 Year (2)	50	14.4 ± 2.9	
15–27 Year (3)	69	15.9 ± 2.6	
Hemodialysis Session and Scale Total Score			
Morning session	97	14.7 ± 2.7	p: 0.03 t: −2.183
Afternoon session	84	15.7 ± 3.0	
Hemodialysis Treatment Year and Scale Total Score^d^			
1–10 Year	74	15.0 ± 3.0	p: 0.04 F: 3.093
11–15 Year	53	14.6 ± 2.9	
15–25 Year	54	15.9 ± 2.5	

*CRF: Chronic Renal Failure*

^a^The difference between the groups 2–3 ^b^The difference between the groups 1–4

^c^The difference between the groups 2–3 ^d^The difference between the groups 2–3

**Fig. 1:**
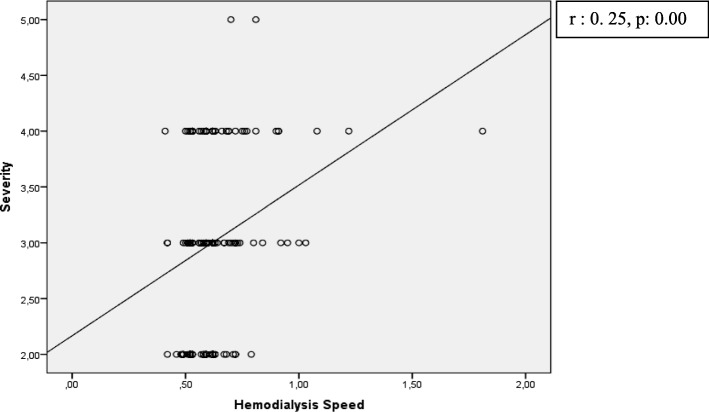
Correlation of hemodialysis speed and 5-D Itch Scale degree (*severity)* dimension

**Fig. 2:**
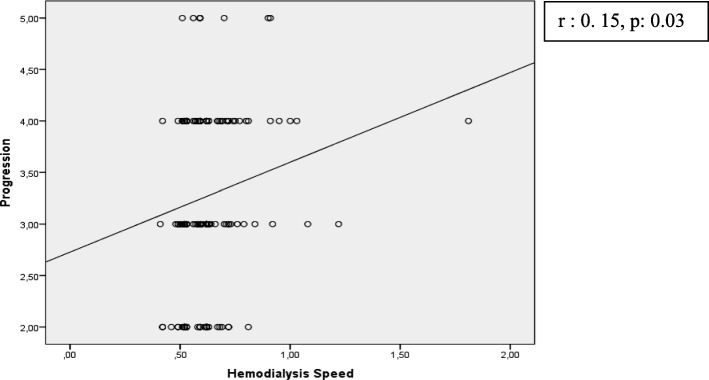
Correlation of hemodialysis speed and 5-D Itch Scale *direction (progression)* dimension

There was no association between the comorbidity of diabetes mellitus and itching characteristics, whereas there was a positive linear relationship between hemodialysis speed and the dimensions of “degree (severity)” and “direction (progression)” (Figs. 1 and 2). As the speed of hemodialysis increased, the severity of itching increased.

## Discussion

Pruritus is the most common complication in patients receiving hemodialysis, but there is limited knowledge on the overall patient symptom experience. The results of this study highlighted the frequency of symptom and the impact on daily life with symptom progression.

In our study, almost half of patients reported itching. The prevalence of pruritus was similar to that in other studies of hemodialysis patients, ranging between 25 and 70% [2, 14–22].

Almost half of patients had daily itching for 6–12 h, while a small percentage had day-long itching. While nocturnal uremic pruritus was commonly reported in a study of 145 hemodialysis patients, 39.3% had day-long symptoms [23]. After 2 days without hemodialysis, itching increased, reaching a peak at night and continuing during dialysis [24]. Similarly, in our study, more than half of patients itched on the day after hemodialysis, possibly because of accumulation of toxins between dialysis sessions. The afternoon session hemodialysis patients reported uremic pruritus with a higher degree, direction, and severity than the morning session patients. The discrepancy between sessions can be explained by clearance during the morning session of toxins and electrolytes that accumulate at night; moreover, more electrolytes may have accumulated in afternoon session patients, resulting in more itching.

Itch degree was defined as moderate or mild by most patients. In other studies in hemodialysis patients, the uremic itch severity score was 6.7 ± 2.4 (on a 10-point visual analogue scale), and the degree was mild in 51.4%, moderate in 11.4%, and severe in 37.7% [14], and moderate (29%) and severe (29%), [25]. Itch degree and total scores were higher in patients aged > 65 years. Similarly, in a study by Weiss et al. in hemodialysis patients, itching was more common in patients aged > 70 years [16]. The skin of elderly patients is drier due to atrophy and decreased hydration in sebaceous and sweat glands [11, 26, 27]. Female patients had higher uremic pruritus scores than male patients, but without a statistically significant difference. The literature on the association between sex and itching is conflicting [4, 6, 14, 18, 21, 23, 27]. Female patients have a higher prevalence of itching than male patients, mostly due to hormonal and psychologic differences. Patients receiving hemodialysis for many years reported more frequent and severe itching. Patients reporting worse uremic pruritus had a longer duration of dialysis than non-itching patients [24]. Differences in prevalence within groups may be associated with accumulation of cytokines and pruritogenic agents over time [28]. In contrast to our findings, Kimata et al. reported that itching was more common in patients whose treatment duration was less than 1 year [20]. It is thought that this may be due to either failure to regulate electrolyte and toxin imbalance, which can cause itching during the first year of hemodialysis, or due to a delay in compliance with treatment.

The direction of itching progression was described as “much better, but still present” by more than one-third of patients. Similarly, in a study by Susel et al., 30% of patients reported ongoing itching [29]. Almost all patients receiving hemodialysis for more than 10 years reported “ongoing itching.” This seemed to indicate that a CRF diagnosis and increase in duration of hemodialysis treatment decreased the likelihood of itching resolution.

Sleep, social/leisure, housework/errands, and work/school areas are affected by uremic pruritus. It is known that itching negatively affects the quality of sleep [6, 30, 31]; more than half of patients in our study reported delays in falling asleep and occasionally woke from sleep due to itching. Similarly, another study reported low sleep quality in hemodialysis patients [20]. Itching caused delays in falling asleep, with sleep interruption at night [20, 27]. In addition to decreasing the quality of sleep at night, day-long itching also triggered fatigue and decreased the quality of life [30, 31]. Short Form-36 Health Survey (SF-36) scores and general health perception among hemodialysis patients with itching were lower, and limitations were reported in social, physical, emotional, and mental functions [6, 29].

In our study, itch distribution was observed in 6–10 anatomic regions (mostly back, upper arm, chest, and abdomen) in 65.2% of patients. In the literature, itching was mostly present on the torso and legs [14] or back and arm [23, 27].

In our study, average Kt/V for hemodialysis adequacy was 1.3 ± 0.16. This is consistent with a target value recommended by a consensus of the National Institutes of Health and Kidney Disease Outcomes Quality Initiative Clinical Practice Guidelines. The recommended Kt/V per hemodialysis session in a patient treated 3 times weekly is 1.4, with a minimum value of 1.2 [32]. Similar to our study, some reports found no significant association between hemodialysis adequacy (Kt/V) and itching [33], while other studies [18, 34] did report an association. According to the findings of our study, as the speed of hemodialysis increases, the degree and direction of itching increases. Urea clearance in hemodialysis treatment depends on the flow velocity of the dialysis solution, i.e., on the speed of hemodialysis. High hemodialysis speed increases the diffusion of urea from the blood to the dialysate liquid [35]. However, this effect does not occur often and can explain the increase in itch severity with low speed. This may indicate that the effect of electrolyte imbalance, which may not be important in the short term, could be critical in the long term.

This study has several limitations. In our study, almost all patients answered the question about disability due to itching at work/school as “not applicable.” As almost all patients were unemployed, comments were unavailable for the effect on work/school life. Studies should also be conducted in patients who are employed and have itching. This study was conducted in 3 dialysis centers of 1 university hospital, where all care was provided in a standard manner. Multicenter research with different care standards should be performed to confirm our findings. In this study, itching was measured once. Studies with repeated measurements might be useful for comprehensive assessment. Other cutaneous manifestations were not assessed in this study, and future research should evaluate these.

## Conclusions

This study is relevant for the scientific community because uremic pruritus assessment included biopsychosocial components based on a comprehensive multidimensional method.

In this study, almost half of the patients had moderate itching. Most patients had ongoing itching symptoms for at least one-fourth of the day. Itching negatively affects the quality of life and sleep. Itching was more common in patients aged > 65 years, those in hemodialysis for > 15 years, and those in afternoon sessions. In addition to studies on the degree of itching, more research is needed on itch severity, impact on activities of daily living and social life, and changes in quality of life and sleep in terms of multidimensional approach. According to our study, health care professionals should consider elderly hemodialysis patients as a high-risk group for itching, and should assess quality of life and sleep in all patients.

## Abbreviation

CRF: Chronic Renal Failure
